# Psychosocial support for parents of extremely preterm infants in neonatal intensive care: a qualitative interview study

**DOI:** 10.1186/s40359-019-0354-4

**Published:** 2019-11-29

**Authors:** Anna Bry, Helena Wigert

**Affiliations:** 1 Division of Neonatology, Sahlgrenska University Hospital, Gothenburg, Sweden; 2 Institute of Health and Care Sciences, University of Gothenburg, Gothenburg, Sweden

**Keywords:** Extreme prematurity, Neonatal intensive care, Psychosocial needs, Parental stress, Emotional support, Nursing

## Abstract

**Background:**

Extremely premature infants (those born before 28 weeks’ gestational age) are highly immature, requiring months of care at a neonatal intensive care unit (NICU). For parents, their child’s grave medical condition and prolonged hospitalization are stressful and psychologically disruptive. This study aimed at exploring the needs of psychosocial support of parents of extremely premature infants, and how the NICU as an organization and its staff meets or fails to meet these needs.

**Method:**

Sixteen open-ended interviews were conducted with 27 parents after their infant’s discharge from the NICU. Inductive content analysis was performed.

**Results:**

Four themes were identified: Emotional support (with subthemes Empathic treatment by staff, Other parents as a unique source of support, Unclear roles of the various professions); Feeling able to trust the health care provider; Support in balancing time spent with the infant and other responsibilities; Privacy.

Parents of extremely premature infants needed various forms of emotional support at the NICU, including support from staff, professional psychological help and/or companionship with other patients’ parents. Parents were highly variable in their desire to discuss their emotional state with staff. The respective roles of nursing staff, social workers and psychologists in supporting parents emotionally and identifying particularly vulnerable parents appeared unclear. Parents also needed to be able to maintain a solid sense of trust in the NICU and its staff. Poor communication with and among staff, partly due to staff discontinuity, damaged trust. Parents struggled with perceived pressure from staff to be at the hospital more than they could manage and with the limited privacy of the NICU.

**Conclusions:**

The complex and individual psychosocial needs of parents of extremely preterm infants present many challenges for the NICU and its staff. Increasing staffing and improving nurses’ competence in addressing psychosocial aspects of neonatal care would help both nurses and families. Clarifying the roles of different professions in supporting parents and developing their teamwork would lessen the burden on nurses. Communicating with parents about their needs and informing them early in their NICU stay about available support would be essential in helping them cope with their infant’s hospitalization.

## Background

Extremely preterm birth, defined as occurring before 28 weeks of gestational age, is a medical emergency [1]. Like other seriously ill newborns, these infants are admitted at birth to a neonatal intensive care unit (NICU). They need hospital care at least until 36 weeks’ postmenstrual age (i. e. gestational age plus chronological age), or sometimes much longer where medical complications arise [2].

The more premature an infant is, the greater the risk of morbidity and mortality [2]. Long-term outcomes vary widely and are very difficult to predict, adding to the uncertainty experienced by parents early in their child’s life [1]. A Swedish population-based cohort study found that, of children born before 27 weeks’ gestational age, 36.1% had no neurodevelopmental disability at the age of six and a half years, while 30.4% percent had a mild disability and 33.6% had a moderate or severe disability such as cerebral palsy, cognitive deficits or visual or auditory impairment [3].

### Family-centred care and parental bonding

In recent years, many NICUs have adopted a policy of family-centred care [4]. The goal of this philosophy of care is a cooperative relationship between the family and the health care provider, where the family participates in caring for the infant and in decisions affecting him or her, open communication exists between the family and health care provider and families’ individual strengths, concerns and circumstances are respected [4]. Enabling and encouraging parents to be with their child at the hospital is one concrete part of implementing family-centred care. In Sweden, parents can receive economic compensation for loss of earnings while their premature infant is hospitalized, allowing both parents to take time off work to be with their infant [5].

It is particularly important for parents at the NICU to participate in the care of their child not only so that they will know how to care for the infant after discharge from the hospital, but also to promote parents’ emotional bonding with the infant. Several factors inherent in the infant’s condition and the circumstances of care in the NICU risk disturbing this process [6, 7]. The infant is separated from the mother at birth and placed in an incubator, and needs extremely careful handling [8]. Parents are dependent on NICU staff for access to their infant as well as for information and instructions concerning his or her care, which can make them feel as though the infant were not really theirs [9]. The fact that extremely premature infants are very different from normal newborns in appearance and behaviour can also impair the formation of parent–child bonds [8]. Early impairments in the interaction between extremely premature infants and their parents, as well as parental posttraumatic stress after the child’s birth, may have long-term adverse consequences for the attachment relationship between parent and child [10, 11].

### Previous research on NICU parents’ psychosocial needs

The birth of an extremely preterm infant is usually an unexpected event for the parents and severely disrupts their expectations surrounding the birth of their child. The stressfulness of having a child hospitalized at the NICU has been well-established in research [6]. Among the major sources of stress and anxiety for parents are fear for their child’s health, alterations in their parental role, the infant’s behaviour and appearance and the highly technical NICU environment [12, 13]. Not uncommonly, parents in the NICU experience severe enough stress to meet diagnostic criteria for acute stress disorder or posttraumatic stress disorder [14, 15]. Other often-reported forms of emotional distress in NICU parents are a sense of loss of control, conflicting feelings of hope and hopelessness and, among mothers, guilt feelings for having been unable to bring their pregnancy to term [6]. Research on psychosocial factors affecting NICU families and their outcomes indicates that severe parental distress during the infant’s NICU stay is associated with worse outcomes both for interaction within the family and for the child’s later cognition and behaviour [1]. Thus, understanding and addressing NICU parents’ psychosocial needs is important not only to alleviate parents’ distress during their child’s hospital stay, but also to contribute to the best possible long-term outcome for the infant and family.

Research on what parents of NICU infants need from the health care provider in order to cope with the psychological difficulties their child’s hospitalization presents has focused attention on several areas of parental need. Information and assurance needs are often considered to be among the most important [16]. That is, parents want to receive clear, open and consistent information concerning their infant and to feel assured that he or she is receiving the best possible care. They also express a strong desire to be close to their infant and empowered to participate in his or her care [17]. Furthermore, studies have described parents’ need for a cooperative relationship with nursing staff, where staff respect them and support them emotionally and in their role as parents [16, 17]. A recent study of parents with critically ill infants in the NICU, an especially vulnerable subset of NICU parents, highlights how vividly the parents experienced both positive and negative interaction with nursing staff [18].

Research to date has largely focused on identifying parental needs rather than explored ways in which these needs are actually met, or not met, at the NICU [19]. Moreover, the psychosocial needs reported by parents of extremely premature infants at the NICU have not specifically been studied.

### Aim

This study therefore aimed to answer the following questions:

- What forms of psychosocial support do parents of extremely preterm infants need from the neonatal intensive care unit (NICU) as an organization and its staff?
- In what ways do they describe the NICU and its staff as meeting these needs or failing to meet them?

## Method

### Participants

Participants were recruited among parents whose children had been born extremely prematurely and hospitalized at a neonatal intensive care unit (NICU) at a university hospital in Sweden. This particular unit has 22 beds and a staff of about 120, including doctors, registered nurses and nursing assistants.

Potential participants were informed about the study at their follow-up visit to the NICU after their child’s discharge from the hospital. Sixteen families were contacted, and all gave their written informed consent to participation in the study. The following exclusion criteria were applied: parents whose child had died, except in the case of twin births where one of the twins had died; parents whose child had been referred to habilitative services owing to severe disability; parents who were not fluent in Swedish.

The gestational ages of the participants’ infants ranged from 23 weeks and 5 days to 27 weeks and 6 days (median 26 weeks and 2 days)*.* The infants had been hospitalized for a total of seven to 13 weeks (median 9 weeks). They had spent between three and 11 weeks (median 4.5 weeks) in a level III NICU (intensive care), and subsequently up to eight weeks (range 0–8 weeks, median 4.25 weeks) in a level II NICU (special care nursery) [20].

Three of the mothers had given birth to twins. In two of these cases one of the twins had died, one at birth and the other at the age of three days.

Eighteen of the participants, representing nine couples, were first-time parents. The remaining participants had one or more older children at home. The participants’ ages ranged from 20 to 47 (median 30). Eleven participants from six families were foreign-born.

Participating families were permitted to decide whether one or both of the parents would be interviewed. In eleven families, both the mother and father participated, while in five families only the mother participated.

### Procedure

Sixteen open-ended interviews were conducted in a place of the participants’ choice; all participants chose to be interviewed in their homes. At the time of the interviews, between one week and four months (median 1 month and 3 weeks) had passed since the infant’s discharge from the hospital, and the infants were between 2.5 and 7 months old (median 5 months). The interviews lasted between 25 and 77 min. Each interview began by asking the parents to describe their experiences of being a parent at the NICU. Follow-up questions adapted to each participant’s account were asked as appropriate, with a focus on parents’ experience of their child’s stay at the NICU, emotional issues that had arisen for them during that period, the sources of psychosocial support that had been available to them and their views on its extent and quality (see Additional file 1: Interview guide). Examples of follow-up questions included: “What helped you cope with the stresses involved in being a parent at the NICU?”; “In what ways did the NICU staff respond to your needs as a patient’s parent?”; “What additional support would you have liked to receive?”

The interviews were audio-recorded and transcribed verbatim.

### Data analysis

The data were analyzed using qualitative content analysis with an inductive approach [21]. The interview transcripts were first read through several times in their entirety in order to obtain a general sense of their content. Meaning units were then identified in the text, condensed and labelled with codes, using the qualitative data analysis software NVivo 11. All data concerning the participants’ experience of their child’s time at the NICU were included in coding as being potentially relevant.

The codes were sorted into subcategories and categories based on their manifest content, after which themes capturing the meaning and implications of the categories in relation to the aims of the study were identified. Research questions were modified and refined during this process to reflect the salient content of the data. Thematization focused on data concerning parents’ descriptions of their needs of psychosocial support from the NICU and its staff, and ways in which the organization and staff had met these needs or failed to meet them. Both authors were involved in this process and reached agreement as to the thematic structure. During thematization, the interview transcripts were continually consulted in order to ensure that the parts were appropriately understood in relation to their context and that important aspects of the data and nuances of meaning were not overlooked in the analysis.

## Results

Four themes elucidating participants’ need of psychosocial support during their child’s hospitalization, and the ways in which the NICU and its staff had met their needs or failed to meet them, were identified in the data (Table 1).

**Table 1:** Themes and subthemes

Theme	Subthemes
1. Emotional support	*Empathic treatment by staff*
	*Other parents as a unique source of support*
	*Unclear roles of the various professions*
2. Feeling able to trust the health care provider	
3. Support in balancing time spent with the infant and other responsibilities	
4. Privacy	

The number after each quotation below is the number assigned to the source interview (i. e. the mother and father in each family, where applicable, have the same number).

### Theme 1: emotional support

#### Subtheme: empathic treatment by staff

Participants expressed a need for emotional support from staff. This support sometimes took the form of explicit consolation or conversations about how the parents were feeling, but could also be transmitted simply through an empathic tone in communication between staff and parents, or through positive attention from the staff.

The participants described the NICU staff as generally kind and caring, and said that staff often provided support and consolation when they saw that parents were suffering. Some participants mentioned that encouraging and hopeful comments from staff had meant a great deal to them.

*“There was a nurse* [who] *said, ‘I’ve seen a lot of babies, I’ve worked with them for many years. Sometimes people think it isn’t worth investing that much in the baby because it won’t make it, but I know* [yours] *will make it and you shouldn’t be worried’. /…/ Yes, it did make me much less worried.”* (Father 8).

At the same time, the participants felt a need for staff to be honest and realistic, not glossing over real problems affecting their infant. They said they needed to feel that their worry and emotional pain were accepted and validated by staff, not minimized or passed over as if their expressions of emotion were invisible or undesirable.

Some participants described a need to have staff inquire directly into their emotional state. When staff showed an interest in their feelings, it made them feel that the staff cared about their needs as well as the infant’s, and gave parents an opportunity of seeking emotional support. Some participants said staff had frequently asked them how they were, whereas others said that such questions from staff were rare. Participants interpreted the absence of such questions in various ways. Some thought the staff did not see it as part of their job to ask parents about their feelings, or that they were too busy to do so, whereas others suspected that staff avoided the subject. In other words, participants pointed to both personal qualities of staff and organizational issues as reasons why they received less support than they would have liked.

*“*[The intensive care unit] *is where you come first, and that’s where the first existential questions come /…/ In the beginning you have an awful lot of questions, a lot of thoughts and you maybe need a lot of attention as a parent. And you don’t get it, and it’s because there aren’t enough staff”* (Mother 16).

Some participants had a sense that staff had paid little attention to them or seemed to downplay their emotional struggles because their child’s medical condition was relatively unproblematic compared to that of some other patients. They pointed out that even if their child was not in danger they were still in an emotionally difficult situation as parents and needed empathy and attention from staff.

*“I was super scared /…/ and I know I asked, is [my baby] strong, or does she seem healthy, or something like that. And* [a nurse] *looked – and it was well meant but it came out really wrong for me, because she said, she almost just snorted at me and said ‘ah, she weighs over a kilogram’ /…/ since then I’ve understood that she was big for her* [gestational age] */…/ but it’s not as though I knew that.”* (Mother 3).

They felt that staff sometimes failed to meet this need, coming across as insufficiently sensitive to the fact that situations that were routine for them as professionals were often stressful and bewildering for patients’ parents. Besides, participants observed that parents at the NICU tended to be in an unusually sensitive state of mind where small gestures from staff that communicated empathy or the lack of it could affect them deeply. Some couples also considered that the father had received less attention from staff than the mother or that staff had seen him as less important to his child than the mother.

Other participants, however, said they felt no need or wish to share their emotional situation with staff. Some developed special relationships with particular staff members and might disclose their feelings to them, but not to others. Some participants felt that talking about their emotions while at the NICU would have made it harder rather than easier for them to cope, and that they needed to put off processing the experience until a later time when they felt more stable. In other words, they needed staff to respect their reserve.

*“The social worker /…/ started to ask, how are things and so on? ‘Yes, well, it’s fine.’ Then she started to probe a bit more and then it just, like, cracked. ‘You don’t want this, do you?’ ‘No, not right now. I’m not up to it.’ /…/ I felt that if I were to let go of… the wall, or whatever, that I had built up, or this defence, I wouldn’t have been able to be there for* [my child] */…/* [sob] *it would have meant going and lying down and never getting up again, pretty much”* (Mother 12).

The participants also expressed a need for emotional support from staff in the form of an empathic and humane style of communication, irrespective of what the topic of conversation was. For example, they needed medical information to be conveyed in a way that showed sensitivity to its emotional effect. Some participants also noted how disconcerting inconsistent messages from different staff members could be for them.

*“There was someone who first… a doctor,* [who explained], *‘well, she* [the baby] *has a vessel that hasn’t closed. It usually closes up when we give this medicine /…/ It doesn’t always help but most likely it will and we won’t need to do anything invasive’. And when he presented it like this I felt pretty calm /…/ Then in the afternoon /…/ there was another doctor who explained it to* [my husband] */…/ she presented it like, ‘yes, it’s a… if the vessel doesn’t close up that means heart surgery and you know, that’s really dangerous for these little ones’…it was like she emphasized all the things there were to be afraid of instead of focusing on the fact that it would probably go well /…/ I remember that was the first time I, like, broke down up there* [at the NICU]*”* (Mother 2).

Participants also noted that information was sometimes difficult to take in when they were tired or emotionally affected. Therefore, they needed staff to take time in conversations with them to allow information to sink in and to give parents a chance to ask follow-up questions, as opposed to giving the impression of being in a hurry to go on to something else. Some participants described occasions when unwelcome situations, such as having their child moved to another ward earlier than originally planned, were made easier by the fact that staff validated their feelings and took the time to explain the reasons for what had happened.

#### Subtheme: other parents as a unique source of support

Many participants described the emotional social support they received from other parents on the unit as valuable, even indispensable. These participants said that other NICU parents could understand and sympathize with their thoughts and feelings in a way that was impossible for people without personal experience of having a premature baby.

*“[W]hen I had her* [another mother at the unit] *I suddenly had someone who understood exactly how I felt /…/ you feel really alone when you give birth* [to a premature baby]. *Because the staff don’t understand how I am /.../ even though they tell me, ‘we understand, we understand’. No, you don’t at all, because you haven’t done the same thing as me”* (Mother 9).

Some participants expressed a wish that the NICU as an organization would do more to facilitate contact between patients’ parents, for example by designating times and places where parents could socialize with each other if they wished. They noted that some parents might otherwise not have the energy to initiate contact with other parents spontaneously or might avoid doing so out of fear of intruding. Also, when a family had a room of its own, contact with other parents occurred less frequently.

Several participants mentioned having appreciated the opportunity to meet parents of older children born prematurely thanks to visits to the hospital from the premature baby society, an association formed for the purpose of supporting parents of premature infants. Others said they had been too tired or busy for these activities.

#### Subtheme: unclear roles of the various professions

The participants expressed differing views, often ambivalent ones, as to the appropriate role of the various professions connected with the NICU in providing emotional support to parents.

Usually the participants had met with a social worker at some point during their child’s hospitalization; some perceived this as more or less obligatory. In some cases their meetings with the social worker had mainly concerned practical matters, such as how to apply for social insurance benefits. In other cases participants had also used these sessions to discuss their emotional situation.

Some parents had also seen a hospital psychologist, whereas others said they had felt no need of speaking to a psychologist, did not have time to do so or had never been offered the opportunity. Meetings with psychologists, when they did occur, were described by the participants as involving broader and more thoroughgoing explorations of emotional issues than meetings with social workers. Access to a psychologist tended to be perceived by the participants as a scarce resource that was reserved for parents in severe emotional distress or those who insisted on receiving it, though one couple expressed the opinion that all patients’ parents at the NICU ought to see a psychologist to help them cope.

The participants said it was helpful and gave them a valuable sense of continuity to have a contact person (usually a nurse, in some cases also a doctor) who was designated as the primary staff member they could turn to with their questions and needs as these arose. Having a contact person whom they could keep in touch with was also mentioned as making transitions between wards less disruptive, since it meant that a familiar staff member was keeping track of how the infant and the family were adjusting to the move.

Some participants felt the most appropriate staff member to turn to for emotional support was someone in the nursing staff with whom they had developed a relationship as a natural result of spending time at the unit. On the other hand, some participants said they felt more comfortable talking about their personal problems with someone not involved in the care of their infant and the day-to-day routine of the NICU. Moreover, some participants were of the opinion that extended conversations about parents’ emotional state should not ordinarily be the responsibility of nurses but of social workers or, if available, psychologists, because of their professional training.

Mother: *“I think it would have been better if we could talk to the* [nursing] *staff, because I didn’t think of things and keep the questions in mind until I met the social worker. All my questions came… they just plopped out when the nurses were there.”*

Father: *“But that also depends on what one wants to talk about. If I’m going to talk about how I’m feeling badly, I don’t want to sit there and talk to just any nurse /…/ They don’t have time and maybe they don’t want to and aren’t able to… if someone has training in talking to people it’s better to have those conversations with them.”* (Mother and father 9).

Some participants said that nursing staff, since they met the patients’ parents on a regular basis, could refer them to a social worker or psychologist when they perceived that the parents needed additional support. On the other hand, some participants questioned whether nursing staff could reasonably be expected to be responsible for identifying parents who needed professional psychological help.

Some participants commented that they would have liked to be informed in a more structured way and early on in their contact with the NICU of what kinds of support were available to them and whom they could turn to in each case. This would involve making allowances for the fact that the initial stressfulness of parents’ situation could make it difficult for parents to retain information they were given and act on it.

### Theme 2: feeling able to trust the health care provider

A trusting relationship with staff and the NICU as an organization was another of the psychosocial needs that participants described. Overall, they expressed a general sense of trust in the quality of the care their child had received and the medical competence of the staff. When this trust was impaired, however, the participants said that their worry was exacerbated. The participants’ accounts highlight the fact that they needed to trust not only that individual staff members were reliable, but also that the staff collectively and the NICU as a system functioned adequately.

One aspect of this trust was feeling secure that staff would accurately and thoroughly inform them about changes in their child’s condition and medical procedures to be performed, without parents’ having to ask. At the same time, receiving clear and consistent answers when they did ask questions increased their confidence in staff. They also appreciated being explicitly invited to call from home at any time if they wanted simply to ask how their child was, thereby being able to trust that they were not disturbing staff if they called frequently or at odd hours.

Conversely, experiences of receiving too little information or receiving it too late impinged on participants’ trust in the staff. In severe cases such incidents were described as shaking not only parents’ confidence in staff as such but also their sense of being fully acknowledged as parents by the staff, which in turn adversely affected their ability to feel like parents and connect emotionally to their infant.

*“*[The baby got sick] *in the evening, and they hadn’t called us. We thought he was well, and I got there about 10, 11 the next day for his feeding, and he’s lying there with machines around him, he had an IV in his head. And they just said, yes, a doctor is going to talk to you. I didn’t know anything. /…/ Then I felt I didn’t want him. Because I had nothing left to show him I was his mother, because I didn’t get to decide when he needed me”* (Mother 11).

The participants also needed to be able to trust that staff themselves were fully informed about the child’s condition and what was being done to him or her. Occasional situations aroused doubts in the participants as to how well staff members kept themselves informed, for example when staff were unaware of what had been done to the child, when different staff members gave contradictory information, or when staff asked parents for facts about the child that they could have found in the case notes. Participants also mentioned situations where information did not seem to be transmitted between staff members or between shifts. The participants described such incidents as eroding their confidence in staff and increasing their stress and worry. Some said they developed an uneasy sense that they themselves had to take some degree of responsibility for making sure that staff were keeping themselves informed.

*“I think a lot of people who work there who came in were like /…/ ‘I want to know and I want to be updated’, and they had bits of paper and wrote things down. /…/ But then there were some who were like ‘I don’t know, you’ll have to ask someone else that. You’ll have to check if there’s someone.’ /…/ it makes one worried and we said so too. We were like, we’ll have to start hanging around there the whole time”* (Mother 15).

The participants said it was generally easier to trust staff members whom they had regular contact with than unfamiliar staff. Thus, frequent changes in staffing could make it harder for parents to go home feeling secure that their child was receiving the best possible care. Some participants also commented that staff discontinuity impaired communication with staff, even tending to discourage them from asking questions in the first place, adding to parents’ sense of insecurity and dependence.

Some individual staff members were perceived as more conscientious and more committed to their patients, and thus more trustworthy, than others. One factor that participants mentioned as contributing to their trust in staff was perceiving staff as having a warm and affectionate attitude towards their child, as opposed to merely seeing to the child’s physical needs.

*“[I]t felt more comfortable going home when certain people were on the night shift, it felt like I slept better because they – I know they’ll take care of my child, you know… it felt like they liked my child /…/ the ones that maybe talked in a sweet way and had some, well… empathy or that talked the way you would talk to your own baby /…/ it makes a really big difference”* (Mother 2).

Trust was also necessary for parents to feel they could afford to speak up if they were dissatisfied with something a staff member had done. This was not always the case; some participants said they had been reluctant to give negative feedback to staff for fear of being singled out by staff as difficult parents. In addition, participants said they had an acute sense of being dependent on the NICU staff for the care of their child, and feared that criticizing staff might have an adverse impact on the care their child received.

Mother: *“I suggested I should talk to her* [a nurse, about an intrusion of privacy] *but* [father, name] *said they’re holding our child hostage, so we can’t.”*

Father: *“Yes, I don’t think we should say bad things about the people taking care of our child because then they might take worse care of her, maybe. It’s better just to…”.*

Mother: *“…Grin and bear it.”* (Mother and father 1).

Thus, some participants felt they had nowhere to turn when there was friction between them and staff.

### Theme 3: support in balancing time spent with the infant and other responsibilities

Another recurring theme concerned parents’ need to find a balance between spending time at the unit with their infant and spending time away for rest, care of older children or other responsibilities. Challenges in this area were not confined to parents with older children at home, but these participants described particular difficulties in and stress related to finding a satisfactory balance between time spent at the NICU and time at home. For some, circumstances such as single parenthood or long commutes between home and the hospital contributed to the problem. Many participants felt that the staff in general did not sufficiently take the complexity of their situation as parents into account, instead trying to get them to spend more time at the hospital than was feasible for them. The parents regarded this as unreasonable pressure that intensified their stress and guilt feelings.

*“[W]hen you’re there you feel guilty for not being with your children at home and when you’re at home you feel guilty for not spending all your time at the hospital /…/ and I got some acerbic comments about that too: ‘yeah, it’s definitely best if you’re here the whole time or as much as you possibly can’. /…/ I understand how important closeness is and I want to give my child all the closeness I can, but I can’t do any more”* (Mother 3).

According to some participants, certain staff members had supported their efforts to find a realistic balance in their schedules and accept that they could not do the impossible. Other staff members, however, seemed insensitive to this need, focusing one-sidedly on the desirability of parents’ being at the unit. What were perceived as inconsistent messages coming from different staff members could be disturbing and frustrating for the participants.

Mother: *“*[One nurse] *said, you have to find a solution /…/ have a little schedule to get your daily life to work out /…/ But she was the only one that I talked to, who said anything,* [who said] *that it was okay.”*

Father: *“/---/ She wanted us to find a system that actually worked /…/ It was really important* [to establish a routine]*, but I felt guilty /…/ I felt we were there much too little. Then it made it worse when* [another] *nurse said that now you need to be prepared to be here a lot more, you’ll have to be here in shifts. Okay then.”*

Mother: *“/…/ I was furious because she disturbed our whole daily routine that we had been trying to build up”* (Mother and father 15).

In other cases, participants said their own determination to spend as much time as possible with their infant had led them to the verge of exhaustion. They appreciated having been alerted to this by nursing staff or by another professional such as a hospital psychologist, who had helped them realize that it was permissible and necessary for their coping to fit in other activities, including recreational ones.

On the other hand, one couple felt that staff could have done more to explain to parents why their presence on the unit was important, to motivate them to be there and to offer them concrete support, such as counselling from a mental health professional, to help them cope with the demands that spending time at the NICU entailed.

*“I wish* [we had been given] *some kind of introduction: having a child at* [the NICU], *what does that imply now we’re here? Why is it so important for you to be there? And* [for the explanation] *to be a little clearer so you understand the importance of it and how… ‘and to help you manage, we’re here for you, and there are the contact nurses who… and you’re going to need counselling”.* (Mother 7).

### Theme 4: privacy

The participants described feeling a need for privacy and opportunities for some degree of quiet and separation from staff and other families while at the unit. Generally, they considered that the NICU environment was not conducive to meeting this need.

Some participants had had a room to themselves during at least part of their child’s hospitalization, while others had not. Having a single-family room was seen as enhancing privacy for parents, but not necessarily as guaranteeing it. Some participants complained that staff came and went without regard to what the parents were doing or respect for their privacy. They said this made it harder for the two parents to feel at home, relax and help each other cope by talking in private.

*“We had our own room, but for them* [staff] *the focus is on her* [the child], *not on us. So they can just come in and be like, ‘now she’s going to have her medicine at her next meal, just so you know. Or now she’s going to have an examination in a little while’. Just by coming in and saying that, they directly interrupt you in your conversation /…/ you couldn’t say what you actually wanted to say or really talk properly.”* (Father 9).

On the other hand, some participants pointed out that the need for privacy could have been met at least to some extent by other means than giving the family a room of its own, if there had been more spaces for parents to withdraw to from the ward for a moment of reflection, to express their emotions or simply to be away from the constant presence of other families and staff.

Mother*: “since you don’t get a room* [of your own] */…/ it’s easy to feel that it’s /…/ the hospital’s child, that you’re more in the way /…/.*

Father: *“But I feel there’s some middle ground that’s missing there. As far as creating the right conditions for being there. The way they talk is either you have a family room or you don’t. But* [the problem is] *that there, like, isn’t anywhere to go and cry /…/ or sit down and nurse /…/ or talk to each other.”* (Mother and father 7).

Participants also commented that the lack of peace and quiet for parents on the unit, as well as of sheer physical space, was inconsistent with the goal of encouraging parents to spend as much time as they could on the unit.

*“It’s not arranged in any sense for you to be able to be there. There isn’t even… take a silly thing like the fact that there isn’t even a hook to hang your bag on /…/ So you feel like… okay, this is… the idea is for parents to be there as much as possible, but there isn’t space for anything, for the parent”* (Mother 2).

A particular aspect of the lack of private space that the participants mentioned was involuntary exposure to other families’ problems, which could be emotionally burdensome. For example, overhearing staff talking about other families and the medical condition of their infants, was described as uncomfortable and, in emergency situations, distressing.

*”‘Yes, she* [another family’s child] *is damned sick’, they* [doctors] *were saying /…/ I was sitting with* [my baby] *in my arms, and then* [I] *just* [heard]*… ‘yeah, this one’s in poor shape’. /…/ I was like, hello, hello! I want to put her back in the incubator now, because I just have to get out of here, because I can’t deal with this kind of thing”* (Mother 15).

Some participants reported that they had found it hard at times to be happy about their own child’s progress because they were all too well aware of other families’ worries and sorrows, sometimes to the point of feeling guilty that their own child was doing better. At the same time, medical problems affecting other people’s children aroused fears of potential medical risks that might affect their own child. Some also said that not being able to get away from other parents’ expressions of emotion made it hard for them to concentrate on developing their relationship with their own child.

## Discussion

This study showed that parents of extremely premature infants needed various forms of emotional support at the NICU, which could include support from the staff caring for their child, professional psychological help or companionship with other patients’ parents. Parents also needed to be able to maintain a solid sense of trust in the NICU and its staff. Furthermore, they expressed a need for support in balancing time spent with their infant and other responsibilities, as well as for privacy while at the NICU. While the participants described many positive experiences of receiving psychosocial support, the study also revealed areas of dissatisfaction where parents wished that they had received more support or that the NICU had been better adapted to their needs.

### Emotional support for parents is a demanding task for staff

Responding to parents’ need for emotional support is a delicate task for NICU staff, since it requires attention to parents’ individual qualities, desires and circumstances. Otherwise parents may feel neglected through receiving too little attention or, conversely, intruded on. Staff also need to be careful not to assume a level of emotional distress that parents may not necessarily feel, which would risk making parents feel worse. A previous study has found that nurses tend to perceive the stress levels of parents in the NICU as higher than the parents themselves do [22]. On the other hand, it is important not to miss identifying parents who do need extra emotional support. Previous research reports that nurses can find it difficult to know when parents are in need of support and when they are coping well [23]. The challenges inherent in judging what support parents need and providing such support are compounded if staff themselves are stressed, tired or emotionally depleted [24].

NICU parents’ dependence on staff for the care of their child places them in a vulnerable position which staff need to be aware of when interacting with parents. For example, as seen in the present study, parents may be reluctant to criticize staff because they are afraid it will have a negative impact on their child’s care. At the same time, unfortunately, staff do not necessarily perceive their own behaviour in the same way as parents do and thus may need feedback from parents. For example, in one study, NICU nurses reported giving parents emotional support more frequently than parents reported receiving such support from nurses [25]. More generally, parents in the present study were acutely affected by both positive and negative incidents that might have had much less impact if the parents had been in a less vulnerable emotional condition – for example, insensitive remarks or small failures in transmitting information that staff might see as unimportant. On a more positive note, parents were also sensitive to small gestures of caring from the part of staff. Similar findings as to the importance to parents of signs of kindness and respect from NICU staff have emerged in previous studies [18, 26].

### Defining the roles of different professions

To make the best possible use of the NICU’s resources for giving parents emotional support, it would be important to define the roles of the various professions involved and how they should collaborate as a team to help parents [27, 28]. This could also lessen the stress on nurses caused by uncertainty as to their role and by the sense that supporting parents is a task that competes with caring for the infant [23, 29]. Well-functioning teamwork, with the open and effective communication among team members that it entails, takes time and planning to develop and can be sustained only if it the organization sees it as a priority and systematically allots time for it in the work schedules of staff.

Some participants in our study expressed the opinion that identifying parents in need of professional psychological help is outside the purview of nursing staff. Along similar lines, previous research has noted that when NICU parents’ access to psychiatric care is dependent on the parents’ psychological problems being recognized by non-psychiatric staff, the rate of referrals is low compared to the rate at which psychiatric concerns actually occur in NICU parents [30]. It has been recommended that all NICU parents should be screened early in their child’s hospitalization by a mental health professional connected to the unit to identify parents with high levels or a high risk of emotional distress [28]. This would also signal to parents that experiencing emotional distress in their situation is not abnormal and that help is available if they need it [28].

Peer support from other parents has also been recommended as one level of a psychosocial support system that should be available to all NICU parents [28]. What is usually envisaged is support from trained volunteers with previous experience as NICU parents, such as the premature baby association in the present study. Interestingly, participants in the present study also emphasized the value of informal support from other parents going through the NICU experience at the same time. It is worth exploring how best to give NICU parents opportunities for supportive contact with one another, to the extent that they desire it.

### Nurses need adequate working conditions to respond to parents’ needs

The parents interviewed in the present study felt it was vital for them to be able to trust in the smooth functioning of the NICU and for staff to communicate with them in a comprehensible, thorough and empathic way*.* Previous studies of NICU parents have identified similar needs [16–18, 26]. Organizational factors making it harder for staff to meet these needs merit special consideration. Problems alluded to by parents in the present study included the high workload and insufficient numbers of staff, staff discontinuity and consequent deficiencies in communication among staff. These issues tended to erode parents’ trust in the health care provider their child was dependent on, even while they appreciated the efforts and commitment of individual members of staff. In a previous study on how NICU nurses see their role in relation to patients’ parents [23], nurses similarly reported that a heavy workload at the NICU, problems with staffing and a high proportion of inexperienced nurses were obstacles to supporting parents emotionally, as was insufficient training in this aspect of their profession. Stress due to poor psychosocial working conditions for staff has been found to be a major contributing factor to safety problems in health care [31]; and stress, burnout and an excessive workload in NICU nurses specifically have been shown to have detrimental effects on safety in neonatal care [32–34]. Organizational problems thus need to be addressed for the sake not only of staff but also of the vulnerable infants cared for in the NICU and their families.

### Parents’ presence at the NICU and its limits

Whereas the fact that restricting parents’ access to their child in the NICU causes the parents emotional distress has been amply documented [13], stress and guilt induced in parents by perceived pressure from staff to maximize their presence at the NICU seem not to have been described in previous studies. Conflicting responsibilities and stressors outside the NICU have, however, increasingly been recognized as an important source of stress for NICU parents [22, 30]. As shown by the present study, parents would need support from staff to balance these responsibilities with the goal of closeness to their hospitalized infant, and such support necessitates a realistic view of how much time parents are capable of spending at the NICU. Perhaps more attention should be paid to the quality rather than merely the quantity of the time that NICU parents spend with their infant.

### Parents’ privacy and NICU design

Today, a single-family room layout is increasingly preferred in NICU design [35]. By and large, single-family rooms have been found to increase parental satisfaction and involvement, and to be associated with better medical outcomes than open-bay NICUs with several infants to a larger room [7, 36]. Single-family-room NICUs also have their disadvantages, however. This type of layout can potentially exacerbate the effects of the organizational problems described above, since it increases nurses’ workload and makes interaction among staff more difficult [36, 37]. It also gives parents fewer opportunities for mutually supportive contact with other parents [38] and may even increase parents’ sense of insecurity and stress [39, 40]. Interestingly, the participants in our study did not see their degree of privacy as simply determined by whether or not they had a single-family room. Providing larger and more varied common spaces for parents could give them a greater degree of control over the sights and sounds they are exposed to, as well as a sense that some areas of the NICU are set apart with their needs in mind.

### Methodological considerations

Only parents who left the NICU with a surviving infant were interviewed in the present study. The experiences and psychosocial needs of parents whose extremely premature infant has died may be expected to differ in significant ways from those of the parents who participated in this study, and the support they are actually offered in the NICU may be different in kind and extent. Parents who did not understand Swedish were also excluded from the study. Being dependent on an interpreter for communication with hospital staff could affect parents’ experience of the NICU in ways not picked up by the data in this study.

In our study, parents were interviewed some weeks to months after their infant’s discharge from the NICU, rather than during the NICU stay as in some previous studies of NICU parents’ needs [16, 18, 26]. Some aspects of the parents’ experience may thereby have faded from their memory. On the other hand, having had time to reflect back on their time at the NICU may have helped the parents sort out the most important aspects of their experience and articulate their needs. One noteworthy point in this context is that being interviewed after discharge may have made parents feel freer to disclose critical views of the NICU and its staff [18, 26].

### Suggestions for future research

At present, despite the significance and complexity of psychosocial aspects of neonatal care, NICU staff receive little structured training in this area. In light of the present study, it would be interesting to explore concrete means by which NICU staff, particularly nursing staff who have the most contact with parents, could become better equipped to meet families’ psychosocial needs, for example through education in how to deal with difficult communication situations and support parents emotionally.

## Conclusions

The needs of psychosocial support of parents of extremely preterm infants at the NICU are complex and vary from family to family. It should be recognized that meeting these needs is an important task that presents many challenges for the NICU and its staff. Staff support the parents in various ways, but their time, resources and training in this area are limited. Under these circumstances, the NICU and its staff come short of being able to give parents all the support that they would require. Improving the working conditions of nurses by increasing their number and their competence in addressing psychosocial aspects of neonatal care would help both nurses and families. Clarifying the roles of nursing staff, social workers and psychologists in supporting parents and improving teamwork among these professions would lessen the burden on nursing staff. Listening to parents, communicating with them about their needs and informing them, preferably at the outset of their NICU stay, about the types of support available to them would be essential steps in helping them cope with the stresses of their infant’s prolonged hospitalization.

## Supplementary information

**Additional file 1.** Interview guide.

## Data Availability

The datasets used and/or analysed during the current study are available from the corresponding author on reasonable request.

## Abbreviations

NICU: neonatal intensive care unit
