# Supplementary material for: Psychosocial support for parents of extremely preterm infants in neonatal intensive care: a qualitative interview study
Source: BMC Psychol. 2019 Nov 29;7:76. doi: 10.1186/s40359-019-0354-4 (PMC6883543; doi:10.1186/s40359-019-0354-4)
Supplement: Supplementary file 1 — Additional file 1. Interview guide. [file 40359_2019_354_MOESM1_ESM.docx]

Interview guide

*Initial question*:

Please describe how you, as a parent, experienced your child’s stay at the NICU.

*Follow-up questions posed as appropriate depending on the content of the parents’ narrative:*

What helped you cope with the stresses involved in being a parent at the NICU?

How did your home situation affect your experience?

In what ways did the NICU staff respond to your needs as a patient’s parent?

Did you feel you could talk to staff about how you were feeling? Why or why not?

Did you feel that the staff were interested in your situation and your needs as parents? Why or why not?

What support did you receive from the staff? Could you describe a specific situation?

What support did you receive from the different professions working at the NICU?

What additional support would you have liked to receive?

Was there some way the NICU/its staff failed to meet your needs as parents? If so, how?

What do you think should be done to meet parents’ needs better at the NICU?

How do you think the different professions at the NICU should support parents?
